# Long noncoding RNAs involvement in Epstein-Barr virus infection and tumorigenesis

**DOI:** 10.1186/s12985-020-01308-y

**Published:** 2020-04-09

**Authors:** Jing Zhang, Xiaohan Li, Jingjin Hu, Pengfei Cao, Qijia Yan, Siwei Zhang, Wei Dang, Jianhong Lu

**Affiliations:** 1 NHC Key Laboratory of Carcinogenesis, Department of Pathology, Xiangya Hospital, Central South University, Changsha, 410080 China; 2 The Key Laboratory of Carcinogenesis and Cancer Invasion of the Chinese Ministry of Education, Department of Hematology, Xiangya Hospital, Central South University, Changsha, 410080 China; 3 Department of Microbiology, School of Basic Medical Science, Central South University, Changsha, 410078 China

**Keywords:** Long noncoding RNA, Epstein-Barr virus, Tumorigenesis, Epithelial cancer, Lymphoma

## Abstract

The Epstein-Barr virus (EBV) is a ubiquitous γ-herpesvirus related to various types of cancers, including epithelial nasopharyngeal carcinoma, gastric carcinoma, and lymphoma. Long noncoding RNAs (lncRNAs) are expressed extensively in mammalian cells and play crucial roles in regulating various cellular processes and multiple cancers. Cellular lncRNAs can be differentially expressed induced by EBV infection. The dysregulated lncRNAs probably modulate the host immune response and other biological functions. At present, lncRNAs have been found to be significantly increased or decreased in EBV-infected cells, exosomes and EBV-associated cancers, suggesting their potential function and clinical application as biomarkers. In addition, EBV-encoded lncRNAs, BART and BHLF1 lncRNAs, may play roles in the viral oncogenesis. Analysis of the specific lncRNAs involved in interactions with the EBV machinery will provide information on their potential mechanism of action during multiple steps of EBV tumorigenesis. Here, we review the current knowledge regarding EBV-related lncRNAs and their possible roles in the pathogenesis of EBV-associated cancers.

## Background

Epstein-Barr virus (EBV) is a ubiquitous γ-herpesvirus, which is the first confirmed human tumor virus [1]. EBV is able to establish latent infection in B lymphocytes and other types of cells such as T, NK, and some epithelial cells. Primary EBV infection typically appears in childhood as a symptomless or mild infection. The establishment of viral latency is vital to maintain lifelong persistence of the virus inside the infected cells. In the latent status, EBV exhibits a severely restricted gene expression profile for the purpose of avoiding host immune surveillance [1, 2]. EBV infection is associated with multiple diseases such as Burkett’s lymphoma, nasopharyngeal carcinoma (NPC), gastric cancer (GC), Hodgkin’s disease and breast cancer [1, 2].

In human genomes, fewer than 2% of genes are transcribed into mRNAs [3]. Emerging evidence has shown that many noncoding RNAs (ncRNAs) also play important cellular functions. Long noncoding RNAs (lncRNAs) are defined as RNAs over 200 nucleotides in length without protein-coding ability and have essential functions for a range of biological processes [4]. LncRNAs are mainly the transcribed and spliced products of RNA polymerase II transcription. They are usually 5′ capped and may contain a polyadenylated tail at the 3′ end. LncRNAs have been confirmed to impact cancer initiation, development and progression and utilize a multitude of mechanisms mediated by specific sequences or structural motifs that bind DNA, RNA, or proteins [4, 5]. According to their position relative to neighboring protein-coding genes, lncRNAs are classified as sense, antisense, bidirectional, intronic, or intergenic. The human genome encodes thousands of lncRNAs that were previously considered ‘dark matter’ or ‘junk’ in the genome. However, recent studies have illuminated the roles of lncRNAs, and they are now considered important physiological regulators of cell homeostasis, growth, and differentiation [4, 5]. Now lncRNAs have been shown to play an elegant role in regulating gene expression at various levels, and thus in affecting aspects of cellular homeostasis including proliferation, survival, migration and genomic stability [5–7]. Collective evidence has also identified the important role of lncRNAs in regulating antiviral responses [8, 9].

In structure, lncRNAs are similar to mRNAs. While, unlike mRNAs which are normally cytoplasmic, lncRNAs show various subcellular distributions that can be detected in the nucleus, cytoplasm, or both. Moreover, lncRNAs are usually tissue specific and less conserved than mRNAs [6, 10]. Some lncRNAs interact with the target genes and regulating their expression, thus mediating cell signaling pathways [5, 6, 11]. Current researches indicate that lncRNAs are dysregulated in the infection of viruses. Howerver, the studies of lncRNAs in this field are still in infancy with regard to their functions. Some lncRNAs can serve as scaffolds to regulate the transport or posttranslational modification of proteins, as well as play an important regulatory role during viral infection such as triggering the antiviral reaction [8, 12].

This review highlights lncRNAs associated with EBV infection including EBV-related host lncRNAs and EBV-encoded lncRNAs, focusing especially on their expression patterns and functions.

## Main text

### EBV-regulated host lncRNAs

In recent years, lncRNAs have been shown to functionally exert both positive and negative effects on innate immunity and antiviral regulators. Some host lncRNAs show differential expression during EBV infection (Table 1), indicating their importance in the host response to EBV infection. The aberrantly expressed host lncRNAs, designated as EBV-regulated lncRNAs, can be regulated by or interacted with EBV-encoded proteins and microRNAs [22].

**Table 1:** Cellular lncRNAs regulated by EBV in the infection and associated cancers

lncRNAs	cancer/cell types	regulation status	references
NR2F21	NPC	down	[1﻿3﻿]
FAM95C	NPC	down	[1﻿3﻿]
LINC01106	NPC	down	[1﻿3﻿]
CH507-513H4.6	NPC	up	[1﻿3﻿]
HAP9-AS1	NPC	up	[1﻿3﻿]
CH507-513H4.3	NPC	up	[1﻿3﻿]
RP4-794H19.1	NPC	up	[1﻿3﻿]
LINC00312(NAG7)	NPC	down	[1﻿3﻿]
LOC553103	NPC	down	[14﻿]
APAF1-AS1	NPC	up	[15﻿]
AL359062	NPC	up	[15﻿]
SNHG8	EBVaGC	up	[16﻿]
RP5-1039 K5.19	EBVaGC	up	[17﻿]
TP73-AS1	EBVaGC	up	[17﻿]
MALAT1	NPC	up	[15﻿]
	293-EBV, AGS-EBV	up	[18﻿]
	NPC C666–1	down	[19﻿, 20﻿]
	diffuse large B cell lymphoma	up	
BC200	293-EBV, AGS-EBV, C666–1	up	[18﻿]
LINC00672	293-EBV, AGS-EBV, C666–1	up	[1﻿8﻿]
LINC00982	293-EBV, C666–1	up	[18﻿]
	AGS-EBV	down	[18]
LINC02067	293-EBV, AGS-EBV C666–1	down no change	[18]
LOC100128494	293-EBV	down	[18]
	AGS-EBV, C666–1	up	[18]
LOC100505716	293-EBV, AGS-EBV, C666–1	down	[18]
IGFBP7-AS1	293-EBV	down	[18]
CYTOR	LCL	up	[20]
NORAD	LCL	down	[20]
7SL	LCL and exosomes	up	[21]
H19	LCL and exosomes	up	[21]
H19-AS	LCL and exosomes	up	[21]

*NPC* Nasopharyngeal carcinoma, *EBVaGC* EBV associated gastric carcinoma, *LCL* Lymphoblastoid cell line

### EBV-regulated host lncRNAs may play roles in epithelial carcinomas

EBV infection contributes to a variety of human tumors, including epithelial carcinomas. NPC is a kind of cancer derived from epithelial cells. It is the most common type of head and neck tumor in Asia, especially southern China, and parts of Africa. Because the symptoms are insidious at the onset of NPC, 75–90% of NPC cases are not diagnosed until an advanced stage, which has a high rate of relapse [11, 23, 24]. Moreover, treatment for patients with NPC is not satisfactory, resulting in a low five-year survival rate and a high recurrence rate [24–26]. Considering the current situation, it is important to characterize sensitive and specific biomarkers for the early diagnosis of high-risk individuals as well as the evaluation of NPC therapeutic effects. EBV infection is closely associated with NPC and is present in almost 100% of low or non-differentiated NPC cases [1, 2]. EBV can establish latent infection in NPC that leads to changes in both intracellular metabolism and molecular expression [1, 2, 27]. Therefore, EBV-related pathogenesis, biomarkers and therapeutics for NPC are worth exploring.

Persistent latent EBV infection has been found among almost all NPC patients, indicating its etiological role in the initiation and progression of NPC. It is necessary to identify potential biomarkers for the early-stage diagnosis, progression and prognosis of NPC. According to a previous study by Xiao-xiao Li et al., the application of next-generation sequencing technology to 7 NPC tissue samples and 7 normal nasopharyngeal tissue samples revealed that 2192 lncRNAs were abnormally expressed in NPC and that 62 lncRNAs trans-regulated genes were involved in EBV infection [13]. In particular, several lncRNAs were downregulated, including lncRNA-NR2F2 antisense RNA1 (NR2F21), lncRNA-family with sequence similarity 95 member C (FAM95C), and long intergenic nonprotein coding RNA 1106 (LINC01106), and some were upregulated, including lncRNA-CH507-513H4.6, lncRNA-THAP9 antisense RNA 1 (THAP9-AS1), lncRNACH507-513H4.3 and lncRNA-RP4-794H19.1 [13]. Sixty two genes potentially trans-regulated by the lncRNAs such as CD44 (Hyaluronan/CD44 signaling plays an important role in head and neck squamous cell carcinoma progression) and interleukin 1 receptor associated kinase 1 (IRAK1) are involved in the EBV infection pathway [13]. However, only a few known lncRNAs have been functionally annotated. Therefore, lncRNA-related pathogenesis, biomarkers and therapeutics for NPC caused by EBV are worth further exploring.

Moreover, LINC00312, also known as NPC associated gene 7 (NAG7), is one of the earliest studied lncRNAs and acts as a tumor suppressor gene in NPC [28]. LINC00312 expression was significantly different between NPC epithelium and various noncancerous nasopharyngeal epithelia. LINC00312 can restrict the proliferation of NPC cells and prevent the progression from G1 to S phase in the cell cycle, thus aggravating cell apoptosis. The expression of LINC00312 is negatively related to EBV-encoded noncoding RNA, EBER1 positively correlated with lymph node metastasis of NPC. At the same time, relatively low or high expression of LINC00312 can be used for the distinction of NPC prognosis prediction with lymph node or non-lymphatic metastasis [28]. Therefore, LINC00312, may serve as a potential target in the diagnosis and therapy for EBV associated NPC.

Interestingly, EBV encodes at least 44 mature microRNAs (miRNAs), and these miRNAs can promote the occurrence and progression of many tumors by directly targeting both host genes and viral genes including some lncRNAs which contain miRNA-binding sites [2, 29]. According to a tissue microarray analysis, EBV-miR-BART6-3p can directly target and downregulate lncRNA LOC553103. The overexpression of LOC553103 can promote tumor proliferation, invasion, mesenchymal transition (EMT), and metastasis in vitro and in vivo [14]. The results undoubtedly illustrate the potential application of LOC553103 in the treatment of NPC. The recognition of cross-talk between viral miRNAs and lncRNAs provides novel insight into the influence of EBV infection on tumorigenesis.

Emerging studies show that lncRNAs may be unloaded into the blood by serum extracellular vesicles such as microvesicles and exosomes that are regarded as vehicles of cell-cell communication [5, 30]. The microvesicles transfer viral and cellular factors, particularly in the case of persistent infections such as those of the herpesviruses, and allow the virus to respond to or control the cellular microenvironment, which could be beneficial to both the virus and host. The lncRNAs released into the blood can be quantitatively analyzed [5, 31]. Therefore, lncRNAs released from tumor cells can serve as potential liquid biopsy biomarkers by virtue of their distinct characteristics [5, 30]. For example, the lncRNAs MALAT1, APAF1-AS1, and AL359062 are significantly upregulated in the serum circulation of patients with NPC [15]. These lncRNAs increase in number after NP69 cells (EBV-negative normal nasopharyngeal epithelial cells) are infected with EBV. In addition, the circulating lncRNAs MALAT1, APAF1-AS1, and AL359062 are also associated with poor prognosis in patients with EBV-associated NPC, suggesting their application as biomarkers for the prognasis of NPC [15]. The findings undoubtedly broaden the lncRNAs landscape in NPC and shed light on the roles of lncRNAs, which would be conductive to the comprehensive management of NPC.

Gastric Cancer (GC) is another type of epithelial carcinoma and is the fourth most common cancer worldwide, and the second leading cause of cancer death. GC is a complicated disease with high heterogeneity. EBV is considered to have an important causal role in GC [1, 2, 32]; thus, one kind of GC is EBV-associated gastric carcinoma (EBVaGC), which is ulcerative and mainly occurs in the cardia and gastric body. EBVaGC is a unique subtype of GC, accounting for approximately 10% of all cases of GC. EBV contributes to the deregulation of host lncRNAs that may play a pivotal regulatory role in human GC. The expression of small nucleolar RNA host gene 8 (SNHG8) in cultured EBV positive GC cells is significantly higher than that in noncancerous gastric mucosa cells or EBV-negative gastric cancer tissues [33, 34]. The expression of SNHG8 in EBVaGC is significantly correlated with tumor-node-metastasis (TNM) stage [34]. Further study shows that SNHG8 interacts with EBV genes, including BHLF1, LF3, BHRF1 and BNLF2a. It is predicted that SNHG8 can modulate the expression of TRIM28, EIF4A2, NAP1L1, PLD3, RPL18A, and TRPM7, which may have direct effects on the stomach. The study also demonstrates that the downregulation of SNHG8 inhibits cell growth, arrest the cell cycle and facilitate apoptosis. A recent study by Jing Liu et al. also revealed that the knockdown of SNHG8 with specific shRNAs inhibited cell proliferation and colony formation, arrested the cell cycle in the G0/G1 phase in vitro and suppressed tumor growth in vivo [16]. Thus, SNHG8 may function as an oncogene and participate in the development of EBVaGC, and this knowledge provides information for the treatment and prognosis of EBVaGC.

LncRNAs can play roles through regulating their target genes. It has been previously validated that lncRNAs and microRNAs function as competing endogenous RNAs (ceRNAs) and suppress each other, thus forming a regulatory ceRNA network (lncRNAs-miRNAs-mRNAs) that regulates target mRNAs [5–7]. In a study by Jing-jing Jing et al. [17], the authors used the integration of multilevel expression data and a bioinformatics approach to identify key elements and interactions involved in EBVaGC and to establish a regulatory ceRNA network involving these key elements of EBVaGC. Overlapping genes and regulators such as CXCL10, GDF5, PTGER3, SMAD5, miR-68773p, RP5-1039 K5.19, TP73-AS1, EBV-miR-BART1-3p and EBV-miR-BART22 were observed in diverse regulation networks. EBV-related miRNAs EBV-miR-BART1-3p and EBV-mir-BART22 were shown to regulate CXCL10 and SMAD5. In particular, two unreported lncRNAs, RP5-1039 K5.19 and TP73-AS1, were identified in the ceRNA regulation network, which possibly provides a new avenue for the in-depth study of EBVaGC [17].

In addition, our recent research based on genome-wide RNA sequencing revealed differential lncRNA expression profiles in EBV genome-infected human embryonated kidney cells (293-EBV), and identified the expression patterns of eight lncRNAs (LncRNA-BC200, MALAT1, LINC00672, LINC00982, IGFBP7-AS1, LOC100128494, LINC02067, LOC100505716) in EBV-positive NPC (C666–1) and GC cell lines (AGS-EBV) (Table 1) [18]. This study emphasized lncRNAs-specific functional alterations possibly influenced by EBV infection. We also explored the potential target genes of the lncRNAs. Interesting, the lncRNAs have some potentially common target genes such as eIF4A3, FUS, and UPF1. The exact function and mechanism of lncRNAs involved in EBV tumorigenesis are underway of investigation by us. The databases of lncRNAs in cancers might be connected with tumorigenic phenotypes in the further investigation. However, we should aware the fact that the expression patterns of one lncRNA may be inconsistent in different cancers and cell types (Table 1), implying their different roles in different cancers. The differentially expressed lncRNAs could be also developed as potential biomarkers in the diagnosis and therapy of EBV-associated NPC or GC.

### EBV-regulated host lncRNAs are involved in lymphomagenesis

EBV is latent in B lymphocytes throughout the host lifetime once it invades and cannot be cleared by the host. Latent EBV infection is related to human lymphoid diseases including Burkitt’s lymphoma, Hodgkin’s lymphoma, and posttransplant lymphoproliferative diseases [1]. EBV infection can induce B lymphocyte proliferation, immortalization, or immune escape in vitro and in vivo [1, 2]. The human EBV-transformed lymphoblastoid cell line (LCL), which is obtained by infecting peripheral blood monocular cells (PBMCs) or primary human resting B lymphocytes (RBLs) with EBV derived from the B95–8 cell lines, has been used extensively for human genetic, pharmacogenomic, and immunologic studies.

In a recent study, the lncRNAs and anti-sense RNAs expressed differentially in RBLs infected with B95.8 strain derived EBV were analyzed by RNA-seq [20]. This study provided the dynamic expression patterns of a series of lncRNAs during EBV infection. There are 26 lncRNAs immediately up-regulated and 33 lncRNAs going down immediately upon EBV infection. Some of them may change the expression status with infection time. In the present RNA-seq investigation, CYTOR and NORAD lncRNAs were found to be important for LCL growth and survival [20]. NORAD can bind to proteins involved in DNA replication and repair [35].

According to gene expression analysis of the LCLs, the lncRNAs 7SL, H19, and H19-AS as well as p53 mRNA showed higher expression in the autologous LCL than in the PBMCs [21]. These lncRNAs are implicated in the development of many other tumors, suggesting that they can promote the progression of EBV-induced tumorigenesis. Nine lncRNAs were found in the LCL exosome cargo: 7SL, H19, H19 upstream conserved 1&2, H19 antisense, HAR1B, HOXA6as, NDM29, SNHG5, and Tsix. LncRNAs H19 and H19-AS were enriched in exosomes and their expression was upregulated, while the rest of the lncRNAs were downregulated and present at lower levels than H19 and H19-AS in exosomes. Some studies also suggest that exosomes released by EBV-infected cells may affect tumorigenesis, metastasis, the tumor microenvironment, immune escape and maintenance of viral physiology, in addition to affecting neighboring cells and playing a long-lasting role in infecting cells [10, 30]. Due to the role that LCL exosomal cargo transfer might play in maintaining persistent infection and tumorigenesis, it is important to characterize the exosomal lncRNAs released from LCLs. EBV-regulated lncRNAs may frequently affect the expression of their neighboring genes through looping. It would be interesting to further characterize their roles in LCLs.

It is noticeable that LCLs are not stable cancer cells and the related study may not well reflect lymphomagenesis. Some lncRNAs such as MALAT1 may be expressed unstably with the time point of EBV infection in LCLs [20]. Whereas, MALAT1 upregulation can promote tumorigenesis and immune escape in diffuse large B cell lymphoma, which may be related to EBV infection [19]. MALAT1 is also a tolerance regulator in immunity that has important implications in the induction of tolerogenic dendritic cells and regulatory T cells [36].

### EBV-encoded lncRNAs

The viral infection generally changes the expression of the host lncRNAs, thus playing important regulatory roles on host genes. On the other hand, the lncRNA encoded by a virus can also modulate the expression of host genes which are critical for the infection and pathogenesis of the virus. EBV is recently found to encode its own lncRNAs from the viral genome.

### EBV-encoded BART lncRNAs

In EBV-infected epithelial malignancies, including NPC and GC, the transcription from the BamHI A region of the viral genome is extensive. The alternatively spliced transcripts are called the BamHI A rightward transcripts (BARTs). BARTs are expressed at high levels endogenously. In EBV-infected gastric tumors, 99% of all virally derived polyadenylated transcripts are from BARTs [37]. These transcripts may result in the production of miRNAs or lncRNAs [2, 37, 38]. EBV was the first human virus identified to encode miRNAs that mapped to the BART regions of the genome [2, 39]. Study has revealed that the BART miRNAs were differentially expressed, abundant among epithelial tissue while barely detectable in lymphoid cell lines [39]. The EBV encoded BART miRNAs have been validated to confer a significant and selective growth advantage to EBV positive tumor cells in vivo, including more efficient tumor seeding, larger tumors and higher and rapider mortality [38]. The function of spliced BART lncRNAs are under exploration by researchers. BART lncRNAs are located in the nucleus of EBV-infected cells. The BART lncRNAs knockdown significantly affects the expression of genes associated with cell adhesion, oxidoreductase activity, metastasis of NPC, inflammation, and immunity [40]. BART lncRNA appears to control Pol II at the promoter region and may thus regulate IFN-beta1 and CXCL8 expression in NPC [40].

The BART lncRNAs are also recently found to be regulated by NF-κB signaling in the induction of EBV lytic replication, which is potentially an option for the treatment of EBV-associated carcinomas [11].

Both the BART miRNAs and the spliced or polyadenylated BART nuclear RNAs are derived from the same primary transcripts from the BamHI A region of the viral genome [39, 40]. These BART-derived transcripts are capable of regulating the expression of host cellular genes. The abundance of the BART RNAs and specific splicing patterns contribute to expression of the BART miRNAs. Impressively, Aron R. Marquitz’s team presented the first evidence that the spliced BART transcripts functioned as lncRNAs independently of miRNA formation. They also confirmed that BART lncRNA could function in downregulating specific cellular genes, including tumor suppressors [40].

During latent EBV infection, BART miRNAs down-regulate the some important tumor suppressors by targeting the 3′ UTR of the genes, and thus altering the growth of tumor cells [2, 40]. Aron R. Marquitz’s team revealed that the BART lncRNAs also play a significant role in the transcriptional reprogramming in latent EBV infection. RASA1 is a negative regulator of the RAS oncogene. The tumor suppressor RASA1 was downregulated in response to BART lncRNA expression without any response to the miRNA target gene assay [40]. This implies that it is the lncRNA rather than the miRNAs that is responsible for RASA1 regulation. Distinct function for the different spliced isoforms of the BART lncRNAs remains to be studied.

The RNA-seq analysis suggests BART lncRNAs regulate genes involved in the unfolded protein response (UPR) [41]. BART lncRNAs may have direct or indirect effects on the master regulatory transcription factors that make up the UPR, including XBP1, ATF4 and ATF6, which are likely responsible for the subsequent decrease in stress responsive genes in EBV tumorigenesis. It is possible that the extremely high level of CpG island promoter methylation in EBV-positive gastric carcinomas promotes the silencing of genes. Hence, it is speculated that the BART lncRNAs may have impact on histone regulation or potentially contribute to directing the DNA methylation phenotype. It is clear that several mechanisms of transcriptional regulation have been ascribed to lncRNAs. Taken together, BART lncRNAs can be important for viral oncogenesis. However, continued explorations are still required on how the BART lncRNAs downregulate genes during EBV latency.

### EBV-encoded BHLF1 lncRNAs

BHLF1, a lytic-cycle gene expressed upon the initiation of latency, 1980 nucleotides in length and G-rich, also encodes a long non-coding RNA (lncRNA). BHLF1 has been shown to function as a lncRNA during the viral replicative cycle, where it contributes to the function of the origin of lytic replication (OriLyt) in cis by forming an RNA-DNA hybrid at the site of transcription [42]. The expression of BHLF1 protein depends on the presence of SM, another lytic-cycle protein [37]. BHLF1 lncRNA was localized to nodules only at the surface of viral replication compartments and was not found within the inner replication compartments [37]. According to Richard Park’s report, during late lytic infection, BHLF1 lncRNA contains a series of tandem repeat sequences, which may play structural and scaffolding roles in recruitment of RNA-binding proteins to VINORCs (virus-induced nodular structures). Therefore, BHLF1 lncRNA may help the viral replication.

## Conclusions

**Fig. 1:**
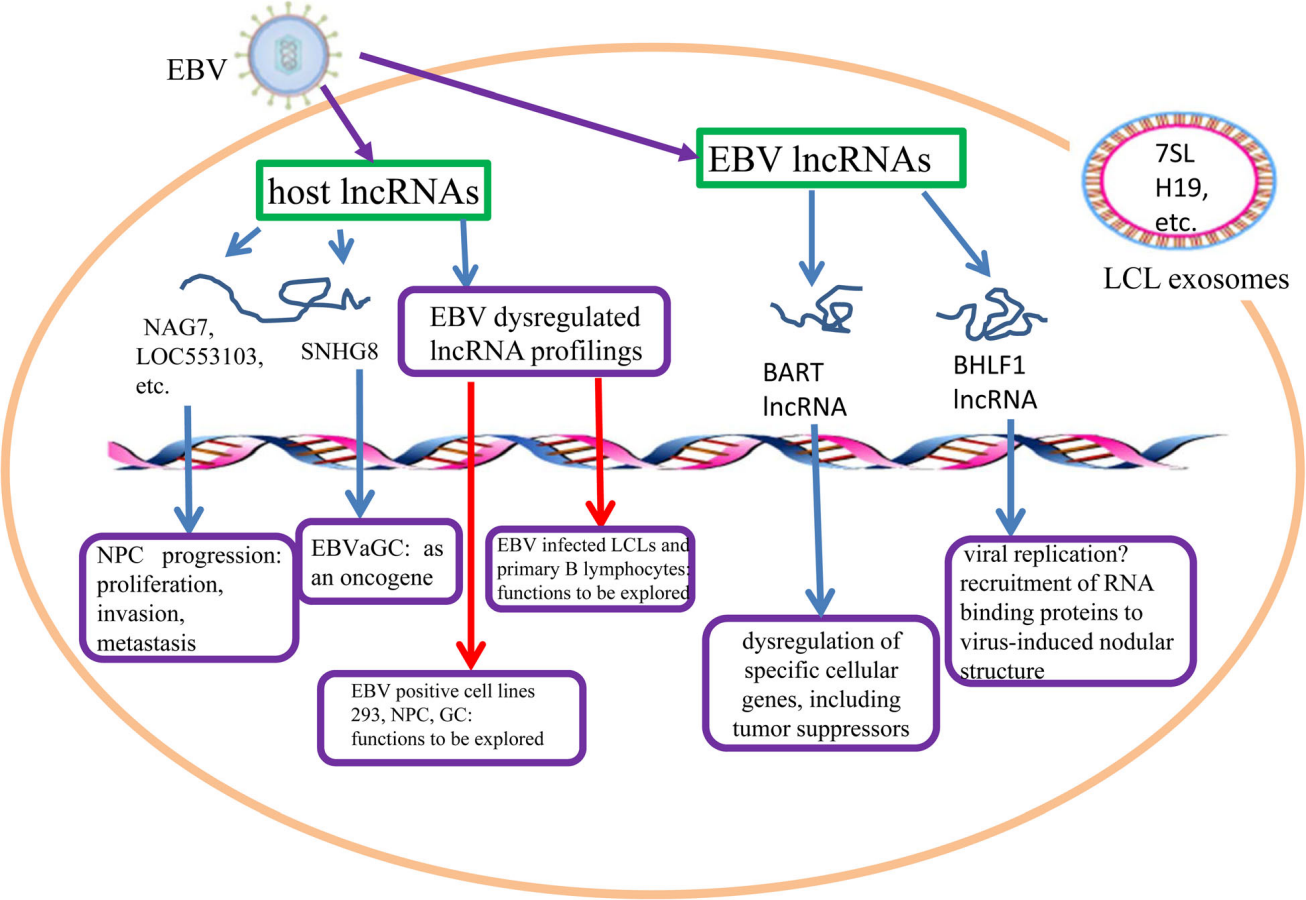
The role of lncRNAs regulated or encoded by EBV. EBV can dysregulate a series of host lncRNAs following its infection, as well express viral lncRNAs. LncRNAs can be involved in cancer progression by targeting cellular functional genes and signaling pathways. The lncRNAs can also play a role in the viral replication to sustain the viral infection and pathogenesis

In summary, cellular lncRNAs are expressed at different levels following EBV infection (Table 1). This is a new insight to help understand the role of viruses in modulating tumor progression (Fig. 1). EBV also encodes its own viral lncRNAs (BART lncRNAs and BHLF1 lncRNA) which may play important roles in oncogenesis and the regulation of some host functions. All the lncRNAs interacting with the host environment during infection potentially improve clinical treatment for EBV infection. In addition, the correlation between lncRNAs and EBV-associated cancers can be detected and analyzed in clinical patient samples. Emerging evidences have revealed the strong regulatory functions of lncRNAs in cancers. LncRNAs are also increasingly regarded as potential biomarkers in the diagnosis, prognosis, and treatment of neoplastic diseases, providing important basic support for medical solutions. However, lncRNAs related to EBV infection and tumorigenesis remain to be further investigated in their functional annotation and our better understanding.

## Data Availability

Not applicable.

## Abbreviations

BHLF1: BamHI H leftward reading frame 1
ceRNAs: competing endogenous RNAs
CYTOR: Cytoskeleton regulator RNA
EBV: Epstein-Barr virus
EBVaGC: Epstein-Barr virus-associated gastric carcinoma
eIF4AIII: eukaryotic translation initiation factor 4A
FAM95C: lncRNA-family with sequence similarity 95 member C
GC: Gastric cancer
GO: Gene Ontology
IRAK1: Interleukin 1 receptor associated kinase 1
KEGG: Kyoto Encyclopedia of Genes and Genomes
LCL: Lymphoblastoid cell line
LF3: Leftward reading frame 3
LINC00672: Long intergenic non-protein coding RNA 672
LINC00982: Long intergenic non-protein coding RNA 982
LINC01106: Long intergenic nonprotein coding RNA 1106
LncRNA: Long-noncoding RNA
LncRNA-BC200: BCYRN1, brain cytoplasmic RNA 1
MALAT1: Metastasis associated lung adenocarcinoma transcript 1
MIR17HG: mir-17-92a-1 cluster host gene
mRNA: messenger RNA
NAG7: NPC associated gene 7
NPC: Nasopharyngeal carcinoma
NR2F21: lncRNA-NR2F2 antisense RNA1
PBMCs: Peripheral blood monocular cells
qRT-PCR: the quantitative real time RT-PCR
THAP9-AS1: lncRNA-THAP9 antisense RNA 1
TNM: Tumor-node-metastasis
UPR: Unfolded protein response
VINORCs: Virus-induced nodular structures
